# Reading, Conducting, and Developing Systematic Review and Individual Patient Data Meta-Analyses in Psychiatry for Treatment Issues

**DOI:** 10.3389/fpsyt.2021.644980

**Published:** 2021-07-29

**Authors:** Nadia Younes, Laurie-Anne Claude, Xavier Paoletti

**Affiliations:** ^1^Université Versailles Saint Quentin, Université Paris Saclay, CESP, Team DevPsy, Villejuif, France; ^2^Centre Hospitalier Versailles, Service Hospitalo-Universitaire de Psychiatrie de l'Adulte et d'Addictologie, Le Chesnay, France; ^3^UFR Sciences de la Santé S Veil, Université Versailles Saint Quentin, Paris Saclay, Gif-sur-Yvette, France; ^4^Institut Curie, Biostatistics, Team Statistical Methods for Precision Medicine, St Cloud, France; ^5^INSERM U900, Statistical Methods for Personalised Medicine Team (STAMPM), St Cloud, France

**Keywords:** synthesis, mediation, heterogeneity, personalised psychiatry, intervention—behavioural

## Abstract

**Introduction:** Individual participant data meta-analyses (IPD-MAs) include the raw data from relevant randomised clinical trials (RCTs) and involve secondary analyses of the data. Performed since the late 1990s, ~50 such meta-analyses have been carried out in psychiatry, mostly in the field of treatment. IPD-MAs are particularly relevant for three objectives: (1) evaluation of the average effect of an intervention by combining effects from all included trials, (2) evaluation of the heterogeneity of an intervention effect and sub-group analyses to approach personalised psychiatry, (3) mediation analysis or surrogacy evaluation to replace a clinical (final) endpoint for the evaluation of new treatments with intermediate or surrogate endpoints. The objective is to describe the interest and the steps of an IPD-MA method applied to the field of psychiatric therapeutic research.

**Method:** The method is described in three steps. First, the identification of the relevant trials with an explicit description of the inclusion/exclusion criteria for the RCT to be incorporated in the IPD-MA and a definition of the intervention, the population, the context and the relevant points (outcomes or moderators). Second, the data management with the standardisation of collected variables and the evaluation and the assessment of the risk-of-bias for each included trial and of the global risk. Third, the statistical analyses and their interpretations, depending on the objective of the meta-analysis. All steps are illustrated with examples in psychiatry for treatment issues, excluding study protocols.

**Conclusion:** The meta-analysis of individual patient data is challenging. Only strong collaborations between all stakeholders can make such a process efficient. An “ecosystem” that includes all stakeholders (questions of interest prioritised by the community, funders, trialists, journal editors, institutions, …) is required. International medical societies can play a central role in favouring the emergence of such communities.

## Introduction

Psychiatry, which focuses on the causation, prevention, diagnosis, and treatment of mental and behavioural disorders, has existed since the late 18th century (1). The true changes that revolutionised psychiatry and shaped current practise date back to the period after the Second World War, with the introduction of standardised diagnoses and the approval of treatments based on evidence. The pharmacological revolution of the 1950s, deinstitutionalisation of psychiatric care for outpatient treatment, behavioural and cognitive approaches in the 1960s (2), development of the DSM-III diagnostic approach, and third-generation cognitive-behavioural approaches (3, 4) have all contributed to the emergence of modern psychiatry. Significant progress has been made in the characterisation of diagnostic entities and their evolution, prevention, and treatment, which now include various forms of psychotherapy, medication, psychosocial intervention, and biological treatment. Nevertheless, both the diagnosis and choice of treatment rely solely on clinical variables and our knowledge about the pathophysiology of mental disorders is still poor (5).

Here, we focus on treatments that are central to current care in psychiatry. The commitment to evidence-based-medicine has led to progress in psychiatry. Many randomised controlled trials (RCTs), considered to be the most authoritative method for evaluating interventions, have been conducted to determine the efficacy, effectiveness, and moderators of treatment effects (6, 7). RCTs are central to the assessment of medications or interventions but they are not able to always address all issues concerning treatments, which are becoming increasingly complex. In addition, conclusions are sometimes based on weak evidence, as single studies are often not replicated (8) and thus the use of certain drugs and drug combinations may be approved without sufficient data. In psychotherapy, the tradition of proclaiming a new method of therapy and backing it up based on clinical experience alone is still common (9). Specific populations have not been or could not be sufficiently explored in RCTs, such as children and adolescents, pregnant women, or populations with a comorbidity. This is also true for many questions encountered in daily clinical practise, such as polypharmacy (10), resistance or difficult to treat disorders (11), long-term approaches and maintenance treatment (12), and compulsory care (13), as well as complex concepts, such as quality of life, recovery, or empowerment. Personalised psychiatry, i.e., considering the sociodemographic and clinical characteristics of a patient to select the treatment of choice, needs to be carefully developed to limit personalisation in a haphazard, inconsistent, and trial-and-error manner, and requires that the knowledge and experience of the psychiatrist is systematic and empirically supported (14). We are even farther away from a form of precision psychiatry that uses objective measures derived from genetics, blood-markers, imaging, or neuropsychological tests to choose treatment (15). Such approaches for treatment and prevention would be able to take into account each person's variability in terms of their clinical characteristics, genes, environment, and lifestyle (5). Thus, it is often difficult to rationally decide what treatment to initiate for a particular patient (and at what point in his or her life), that is compatible with his/her entourage and resources for a particular clinical presentation that may include certain comorbidities. This has led to predominant pessimism about the actual feasibility of the personalisation of treatment of depression in routine clinical practise (7). The data are often still too scarce to guide the choice of treatment based on any one of these aspects, and are too incomplete to address chronic or recurrent disease and compliance and implementation issues in the long term.

Meta-analyses (MAs) consist of the secondary analyses of RCTs databases with several objectives but above all, to provide an unbiased and accurate estimate of an intervention effect that can be extrapolated to the population under study. There are two types of MA: (1) those that use aggregate data (AD) retrieved from the published literature or from study authors to quantify the relative efficacy or safety of a treatment and (2) individual participant data meta-analyses (IPD-MAs), the focus in this article, which include the secondary analyses of the raw data from databases of relevant clinical trials (ideally), identified through a systematic review, after a certain amount of standardisation (16, 17). Many MAs from aggregate data have been published in psychiatry, whereas only ~50 IPD-Mas have been performed since the late 1990s, mostly in the field of treatments. A 2015 review showed that the IPD method has been primarily applied to cancer and cardiovascular disease but appears to be gaining popularity in other fields, such as mental health. It also established that only 1% of the MAs conducted were IPD-MAs (18).

In this paper, we review the various steps to conduct and read IPD-MAs exploring treatment-related questions in the context of psychiatric diseases. The PRISMA IPD guidelines (19) and the material published by the COCHRANE Collaboration IPD Methods Group provide helpful resources. We provide guidelines for the identification of the relevant trials, the preparation and standardisation of data, and their statistical analyses and interpretation. All steps are illustrated with examples in psychiatry for treatment issues. We conclude with some recommendations on the organisation of MA groups to obtain a better involvement of all healthcare stakeholders.

## Some Objectives of IPD Meta-Analysis

Interventions cover many therapeutic aspects, such as the effect of a class of psychotropic drug, psychotherapy, the quality of healthcare, the follow-up of patients, etc. From here on, we will use the generic term “treatment.” IPD MAs are particularly relevant in psychiatry for treatment issues for the following objectives.

(1) Evaluation of the average effect of an intervention

The most common objective is to combine the effects from all trials that addressed a similar question to estimate an overall treatment effect with high accuracy. Such an overall effect results from the “true” mean effect, inter-trial fluctuations, and possible biases. The statistical power of combining several databases allows the detection (or exclusion) of even mild effects. This is particularly necessary if the conclusions of the trials are discordant. The average treatment effect is closely related to the field of Public Heath, in the sense that it applies to a large number of patients, and is considered to be the highest level of evidence (20). It is important to highlight that the primary purpose of MA is to quantify the treatment effect with its confidence interval, and not to test a null hypothesis. Due to the high statistical power provided by the large amount of data, non-clinically significant effect may appear statistically significant. Clinical judgement is then central for the interpretation of MA results. IPD-MAs apply to different types of therapeutic interventions and different average effects, as illustrated in examples 1–4 in Supplementary Material 1.

However, although healthcare authorities may base recommendations on such average effects, the data may be insufficient for practitioners, who consider various patient characteristics to decide upon the best therapeutic approach. In addition, true effects may be quite variable across various sub-groups of the population as such an average effect combines the evidence from different RCTs that have been carried out on different populations with different healthcare systems and different eligibility criteria or a different case mix.

(2) Evaluation of the heterogeneity of an intervention effect and sub-group analyses

Indeed, the average effect results from a mixture of several situations. Although a certain effect may be expected for all patients, the same average effect may involve a mixture of patients who do not benefit at all from the intervention and some who strongly do; this is called heterogeneity. For example, outcomes during treatment with antidepressants or antipsychotics metabolised by cytochrome P450 (CYP) 2D6 are heterogeneous, because the effects are heterogeneous due to the varying activity of this enzyme among individuals, ranging from poor (adverse effects) to normal or ultrarapid metabolism (non-response) due to polymorphism of the CYP2D6 gene. The dose of antidepressants has to thus be adapted (21, 22). Detecting and exploring heterogeneity is an important component of IPD MAs that offers unique opportunities and benefits when investigating effects on subgroups (23). Large sample sizes and numerous trials allow for the assessment of treatment effects adjusted for various characteristics, i.e., the treatment effect can be estimated in subgroups of patients to approach personalised psychiatry. Subgroups can be investigated (i) at the trial level (e.g., geographical regions, time period etc.), which is also called meta-regression, or (ii) at the patient level, based on individual patient characteristics (e.g., age, initial symptom severity, etc.). Such characteristics can be either prognostic (i.e., associated with outcome regardless of the treatment) or treatment effect modifiers (variables associated with differential responses depending on the treatment). Most RCTs lack the power to evaluate subgroups, as the sample size rapidly decreases when slicing the population into subgroups. Furthermore, the investigation of subgroups leads to multiple tests, which increases the type I error rate (false-positive rate). Only meta-analyses provides adequate control of the risk of errors due to their large sample sizes. Several studies have been conducted with this objective: For instance, to determine whether initial severity or individual symptoms modulate the efficacy of antidepressants for major depression, anxiety disorders, OCD, or PTSD or that of antipsychotics efficacy (24–27). Other examples are provided in examples 5 and 6 (see Supplementary Material).

(3) Mediation analysis and surrogacy evaluation

In multifactorial and complex chronic psychiatric diseases, interventions may affect the course of the disease or adverse events at various stages evaluated by different endpoints (9, 28). The overall evaluation of the intervention thus combines so-called intermediate endpoints (as response) and final endpoints (e.g., remission, recovery). A question of central interest is whether the effect of an intervention on the final outcome is direct or mediated by the effect on intermediate endpoints and the relative weight of each effect. Mediation analysis is a sophisticated statistical analysis to disentangle each component that only IPD-MAs can provide. Such analyses provide unique insights into (i) the effect of an intervention on the various endpoints and (ii) the interrelation between the various scales used to assess endpoints as illustrated in example 7 (see Supplementary Material). It would be interesting to develop this approach in psychiatry for final outcomes.

A surrogate endpoint could also be intended to replace a clinical (final) endpoint for the evaluation of new treatments when it can be measured more conveniently, more frequently, or earlier than the final endpoint. It is expected to predict clinical benefit, harm, or their absence. To statistically validate surrogate endpoints, the meta-analytical approach is presented as the gold standard in the literature, in particular in oncology (29). Two levels of surrogacy are classically distinguished (29): that at the individual level and that at the trial level. The first addresses the question of whether a high/low value of the intermediate endpoint entails a high/low value of the final endpoint for a given patient. The second quantifies whether a treatment effect on the surrogate endpoint (e.g., the symptoms) entails a treatment effect on the final endpoints. In other words, a patient can have less severe disease, as measured at the intermediate endpoint, and a better final outcome, but this does not necessarily mean that modification of the intermediate endpoint by the intervention will lead to a better final outcome (the intervention only influences the intermediate endpoint, not the final one). Although it is relatively simple to evaluate the correlation between two endpoints at the patient level in single trials, only meta-analysis of several trials allows the quantification of trial-level surrogacy. This approach would provide important information if it were more widespread in psychiatry, for which there are many studies with follow-up for efficacy or adverse events (such as weight gain). The example 8 (Supplementary Material) gives an illustration of possible results.

## Methods

### Systematic Review and Trial Identification

As for any clinical research, a protocol for the IPD-MA must be written and posted on a public registry, such as Prospero (https://www.crd.york.ac.uk/PROSPERO). It lists the objectives and endpoints, the inclusion/ exclusion criteria for the RCT to be incorporated in the MA, and the statistical analyses plan. As IPD-MAs combine evidence from RCTs that received ethical approval from their respective regional ethical review boards, no modification of the informed consent is requested; approval from an institutional ethics board may be requested.

(a) Define the selection criteria

Comprehensive data collection is a central requirement for the evaluation of interventions to avoid selection bias. Certain data can be assessed directly from published information; other studies require patient-level data (such as population characteristics, modality of the intervention, and quality of the trial). The requested variables can be grouped into three sets:

#### Intervention

The intervention must be specified and standardised as much as possible to be comparable across RCTs: doses or treatment duration, therapeutic principles, professional type or education and the number/duration of sessions, etc. For example, one IPD-MA investigating the efficacy of drug treatment for acute mania across geographic regions restricted the analyses to treatment groups that were given a proven effective dose if the drug was registered for acute manic episode. If it was not registered, an expert consensus agreed on what would constitute an effective dose. A duration of 3-weeks was chosen, following guidelines for clinical investigations of medical products (30). Two other examples (labelled 9 and 10) are provided in Supplementary Material.

#### Population and Context

This includes both the disease definition and the characteristics of the patients. As definitions of disease are regularly refined over time with the advance of knowledge and the emergence of new diagnostic tools, broad criteria should be used. Subgroup analyses, restricted to certain specific characteristics, may be planned. Similarly, it is preferable to enrol a highly heterogeneous case mix of patients and evaluate possible modifications of the response to treatment according to the case mix at the time of the analysis. “Start global and then refine” is a simple guideline.

#### Relevant Endpoints

The endpoints and how they are measured should be defined. In particular, the timing of the assessment, the summary of scales (global score, score of specific dimensions etc.), if some are used, should be described as illustrated in example 10 (Supplementary Material). This aspect is closely related to the issue of standardisation, which is addressed in the next section.

(b) Algorithm Search and Data Sources

A complete algorithm needs be presented in the protocol and reported in any publications. In addition to a literature search using standardised MESH terms, the search is completed with the proceedings of specialty congresses and registries of clinical trials. Not only is a list of all (past or ongoing) trials for given interventions available on clinicaltrials.gov, but certain results are increasingly reported. Registries are less prone to publication biases. A selection of the eligible trials by two independent individuals is recommended. Two examples of trial selection are provided in Supplementary Material 2: one for pharmacological treatments and one for psychotherapies. Flow-charts of the search are presented for illustration.

A comprehensive collection is necessary for the evaluation of an intervention effect to avoid selection biases. For example, certain pharmaceutical companies only release data from trials of approved products; unavailability of data from negative trials entails overestimation of the overall effect in the collected trials. Selection bias is less a concern if the primary question is the evaluation of surrogacy or mediation analyses, as it is unlikely that the availability of IPD depends on the level of correlation between endpoints. Failing to follow those good practise recommendations can limit the findings of a study. As an example, Imai et al. (31) studied an interesting topic: the impact of melancholic features on the response to antidepressants in major depression based only on the data of three clinical trials released by pharmaceutical companies in Japan (31). This limited the amount of included data and the power of the IPD-MA but also the applicability of their results; there is a potential selection bias due to the specific source of data coming from a single geographical area.

Access to IPD is strongly encouraged by most stakeholders. Several journals and funders require a statement on how to access the data for all published clinical trials (32). There is no unique source for data access but some providers are progressively emerging. For example, ClinicalStudyDataRequest.com (CSDR) is a consortium of clinical study sponsors/funders whose goal is to facilitate access to patient-level data from clinical studies.

Data from all patients (analysed and excluded) with the longest possible follow-up is requested; IPD-MAs often have additional data or more complete follow-up data than the source publications, as follow-up update are commonly collected after the first publications of the RCTs. The process of trial selection is represented in the PRISMA flow-chart. The number of studies obtained in relation to the number of studies identified in the research must be indicated. RCTs included and those not included in the IPD-MA should be compared for the endpoints of interest.

For example, Turner et al. conducted an IPD-MA to evaluate the efficacy and moderators of cognitive behavioural therapy for psychosis vs. other psychological interventions (33). They examined whether RCTs included in the IPD-MA differed in post-treatment outcome from RCTs for which they were unable to obtain databases. They corrected for small samples.

### Data Management and Assessment of the Risk of Bias

(a) Standardisation of Collected Variables

For a given intervention, endpoints of each RCT tend to capture the same outcome but assessment (scales, timing) may differ. To some extent, IPD allow standardisation of such information, sometimes after some interpolation as shown in example 5 (Supplementary Material). The same standardisation is performed for baseline characteristics. Staging or severity of the disease, prior therapies, and medical history are homogenised with the intention of limiting missing data, as they will be needed in the analyses of subgroups or to obtain comparable exposure (described in examples 11, 12, and 5 of Supplementary Material). Such standardisation often comes at the cost of a certain reduction in the information. However, the strength of meta-analyses comes from what is shared across trials, not from their multiple specificities.

(b) Assessment of the Risk of Bias

The list of identified trials is further examined to detect possibly biassed trial results. The quality of RCTs is first investigated by rating the risk of bias followed by reanalysis and checking of the IPD. Studies with a high risk of bias should be excluded.

As stated in the CONSORT statement and by the Cochrane Collaboration, the quality of the design of an RCT relies mainly on an adequate randomisation process, concealment of allocation, balanced follow-up, the blinding of intervention, and the management of missing data (34). The Cochrane collaboration has updated this scale to include the assessment of various types of possible biases; in addition to the quality of randomisation, the blinding and the incomplete outcome data, the bias due to selective reporting also called spin has been added; this is an important one, that is however less an issue when the IPD can be obtained (35). The GRADE scale is increasingly used to quantify risk of bias (36). IPD MA allows for more in depth cheques. In fact, the quality of randomisation can be easily assessed on individual patient data. Simple statistical tools have been proposed to detect possible departures from fair randomisation. Such an analysis is carried out for each trial separately and includes the accrual over time, the balance of measured prognostic factors and patient characteristics over time, etc. (19, 37).

Follow-up is also carefully examined. Although the frequency of assessment is not an important concern, it must be similar across compared arms within a given trial. For example, more frequent assessment of the endpoint in the investigational arm than in the control arm results in a very strong bias (37) and should lead to exclusion of the trial.

The evaluation of blinding is more difficult, as very few trials collect data on the quality of blinding (38). It is not uncommon that solely information from the protocol is used.

**Figure 1:**
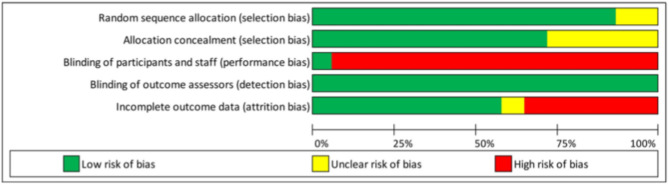
Example of a risk-of-bias representation as reported by Riper et al. (41) (Figure 2).

Another important issue is the management of missing data. A large amount of missing data may strongly affect the validity of the trial. Although certain statistical methods have been proposed to account for missing data (39, 40), they all rely on strong and unverifiable hypotheses. Therefore, high rates of missing data on key endpoints may lead to exclusion of the trial. No threshold has been specifically recommended, but this should be specified upfront. In the IPD-MA assessing the effectiveness and treatment moderators of internet interventions for adult problem drinking presented an assessment of the risk-of-bias of the 19 RCTs, which is reproduced in Table 1 and Figure 1. The quality of the RCTs based on the GRADE score was relatively high. Study dropout was, however, high (over 30%) for seven studies (41) and a high-risk of bias was detected related to the (non) blinding of participants. The latter was expected, as this criterion is difficult to meet for behavioural change trials.

**Table 1:** Example of a risk-of-bias assessment for 19 RCTs as reported by Riper et al. (41) (Supplementary Table 1).

	**Risk of bias**				
**References**	**Random sequence generation**	**Allocation concealment**	**Blinding of participants and staff**	**Blinding of outcome assessors**	**Incomplete outcome data**
Araki et al. (42)	Unclear	Unclear	High	Low	Low
Bertholet et al. (43)	Low	Low	High	Low	Low
Bischof et al. (44)	Low	Low	High	Low	Low
Blankers et al. (45)	Low	Low	High	Low	Low
Boon et al. (46)	Low	Low	High	Low	Low
Brendryen et al. (47)	Low	Unclear	High	Low	High
Brendryen et al. (48)	Low	Unclear	High	Low	High
Cunningham et al. (49)	Low	Unclear	High	Low	Low
Boß et al. (50)	Low	Low	High	Low	Low
Hansen et al. (51)	Low	Unclear	High	Low	High
Hester et al. (52)	Unclear	Unclear	High	Low	Low
Khadjesari et al. (53)	Low	Low	High	Low	Low
Postel et al. (54)	Low	Low	High	Low	High
Riper et al. (55)	Low	Low	High	Low	High
Schulz et al. (56)	Low	Low	High	Low	Unclear
Sinadinovic et al. (57)	Low	Low	High	Low	High
Suffoletto et al. (58)	Low	Low	High	Low	Low
Sundstrom et al. (59)	Low	Low	High	Low	Low
Wallace et al. (60)	Low	Low	Low	Low	High

The size of trials is important, as small sample sizes are commonly associated with a higher risk of bias and selection of studies with a minimum sample size (for instance of more than 50 patients) is sometimes done (61).

Finally, the following reasons for excluding trials should be carefully considered:

(i) Restricted length of the trial: this is often guided by practical concerns, but periods should be long enough not to exclude important trials.
(ii) Language: although it is common practise to focus on trials reported in English, there are no scientific reasons not to consider randomised clinical trials in other languages (A translator may be requested).
(iii) Sponsor type: Intervention trials are classically carried out by pharmaceutical companies, academic collaborative groups, or hospitals. Whether the quality of the trials and outcomes depend on the sponsor is a matter of debate (62). There is no reason to exclude any of them upfront.

### Statistical Analysis and Reporting of the Results

The intent-to-treat principle should apply; all randomised patients should be analysed, whether or not they initiated the treatment and regardless of protocol deviations. The characteristics of included studies and participants (demographic and clinical) are presented. A first summary of the included data (at both the trial and patient level) provides the groundwork for the interpretation and applicability of the full meta-analysis. Statistical analyses thus depend on the primary objective of the meta-analysis.

#### Analyses for the Evaluation of the Average Effect of an Intervention

Patients are compared within the same trial to maintain the benefit from randomisation and obtain a causal effect, i.e., analyses are stratified or adjusted for the trial. Stratified odds ratios or relative risks for binary data, stratified hazard ratios for survival data or adjusted mean effects quantify the overall effect while accounting for the size of each individual RCT. Two estimation approaches are available: (1) In two-stage or fixed-effect meta-analysis, effects from each trial are weighted by the inverse of their variance and (2) in one-stage or random-effect meta-analysis, a random effect of the trial captures the inter-trial variability. The fixed-effect-model assumes that all included studies come from the same population with the same treatment effect; variation are only due to random fluctuations. In practise, this is hardly ever the case as interventions, populations or assessments are not exactly the same. Conversely, in the random-effects-model, the true treatment effect may also vary across studies. The variance of the distribution of true effect sizes, often denoted by τ^2^, is an additional parameter of the analysis. As a result, the two approaches differ in how each trial is weighted to obtain the pooled estimate. In fixed effects MA, the weight is directly proportional to the size of the trial (number of patients or number of events), while in random-effect MA, small studies are given a much larger relative weight. This is a reason why the best approach is a matter of debate. Although both approaches generally yield close results in the absence of strong inter-trial heterogeneity, the one-stage approach is recommended if there is high inter-trial variability, i.e., when intervention effects strongly vary between trials. However, to analyse the data with the two approaches and then to select the one used for the report is discourage as it is data-driven, which increases the risk of bias. The investigation of heterogeneity is an important part of any MA that is introduced in the next sub-section. Forest plots display the intervention effect within individual trials and overall. The effect is estimated without adjusting for any covariates. Both overall and trial-by-trial confidence intervals are represented. The example 13 (Supplementary Material) gives an illustration of a random-effect meta-analysis displayed in a Forest Plot.

#### Analyses of Heterogeneity of the Intervention Effect and Sub-group Analyses

##### Trial Level

Heterogeneity between trials and groups of trials (e.g., defined by different modalities of treatment, follow-up, or evaluation scales) is most often quantified using the *I*^2^ statistic, which represents the proportion of variation of an effect likely due to mere chance. Values above 50% are sometimes used to define strong heterogeneity. In fixed effect MA, the Cochran Q-test statistics, a chi^2^ test, detects variations of the treatment effect beyond that is expected from mere random fluctuations, bearing in mind that tests for heterogeneity may lack statistical power if few trials are included. In random effect MA, τ^2^, the parameter that captures variations intra-trial variability can also be tested, even though the statistical properties of this test are debated as it is often falsely positive. The cause of heterogeneity can be investigated by estimating the pooled treatment effects in subgroups of trials sharing common characteristics. Forest plots are represented for sub-groups of trials as illustrated in example 14 of Supplementary Material; if there is no heterogeneity left in subgroups, and if a difference between subgroups is significant, it may indicate that the subgroup variable was a potential cause of heterogeneity. Of note, if several subgroups are defined according to the treatment, Q-test should not be interpreted as an indirect comparison of the different treatments. Indirect comparisons obtained in the framework of network meta-analyses are not covered in this communication.

##### Patient Level

**Figure 2:**
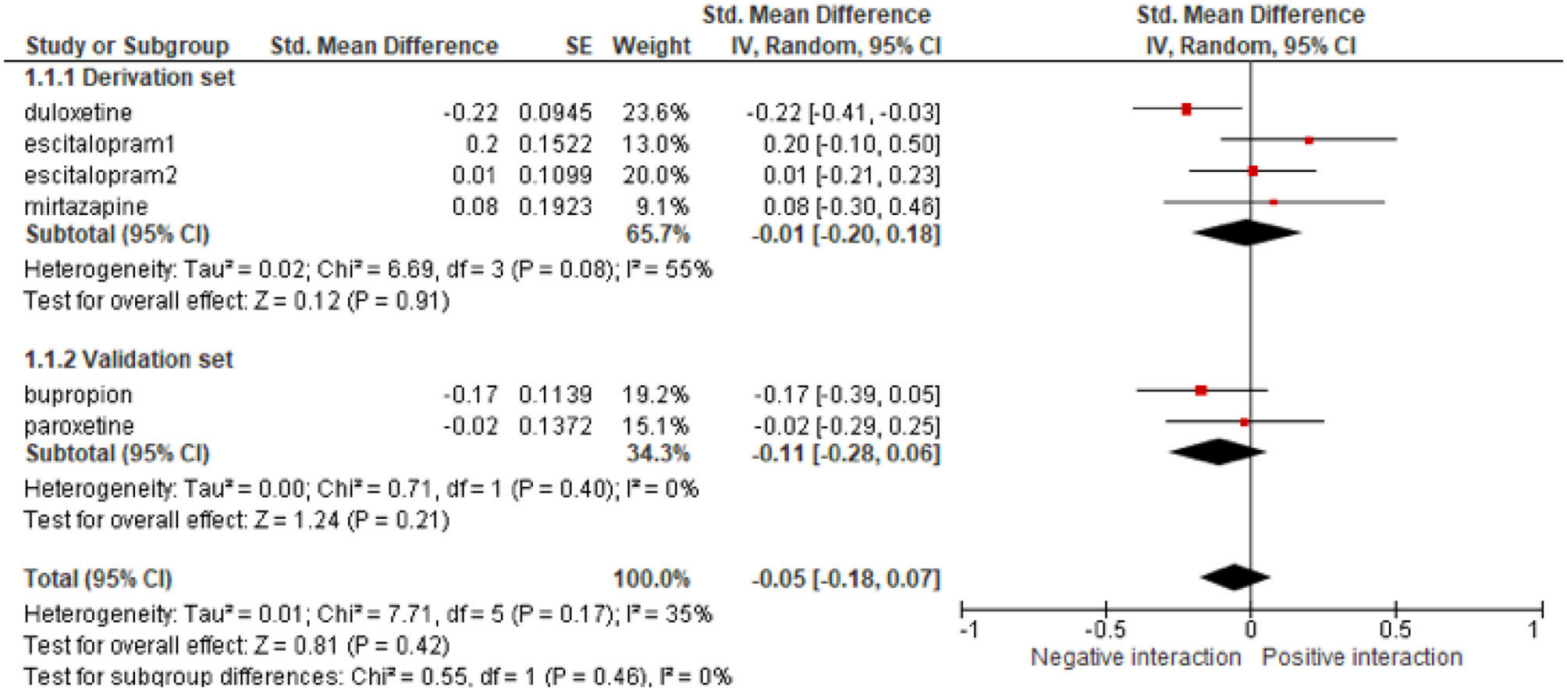
Example of the analysis of the efficacy of anti-depressants at the trial level in subgroups of trials defined by the baseline severity of major depression, reproduced from Furukawa et al. (26). SE, standard error.

The most classic approach to study the effect of an intervention on subgroups defined by patients characteristics (age, medical history etc.) is to build a multivariate model that incorporates the treatment effect, the patient characteristics, and their interactions, while adjusting for the trial. There is however a risk of ecological bias, i.e., a risk of confounding factors due to different case-mix distributions across trials. It is then strongly encouraged to apply the so-called DEFT method that consists in estimating the interaction model in each trial and then to combine the estimates using standard meta-analytic methods (63). For instance, in the IPD-MA on anti-depressant effect, already cited as example (example 14), the authors investigated whether the baseline severity of major depression modified the efficacy of treatment effect. As shown in Figure 2, the estimated the interaction in each study and provided a pooled estimate that supported the conclusion of lack of interaction. A wrong analysis would have been to split data into subgroups, to combine treatment effects within these subgroups, and then compared to compare pooled estimate across subgroups. As pointed out by Fisher, this process combines within-trial and across-trial interaction estimates, entailing a risk of bias (63).

Recently, Vo et al. developed an approach based on causal-inference to “standardise” the case-mix across trials, as if the same population had been enrolled in all of them. This allows investigation of the variation of treatment effects while controlling for possible case-mix heterogeneity (64). It has been developed for binary outcomes. Thus, far, this approach has not been used for IPD-MAs in the field of psychiatry and should be considered for future research.

#### Mediation Analysis and Surrogate Evaluation

A complex model not only relates the intervention to the intermediate endpoints to estimate direct effects, but also the intermediate to the final endpoints to estimate indirect effects. This is represented by direct acyclic graphs, in which the various possible relationships are drawn (see Figure 2). The various mediations can be estimated under certain assumptions. In particular, they imply that the final endpoints do not affect the intermediate endpoints, which is often difficult to establish. In addition, to obtain unbiased estimates, one has to assume that there are no confounders of the symptoms-final outcomes relationships, which are themselves affected by the intervention. Most of the assumptions come from the fact that such analyses are rooted in the causal inference framework, which aims to characterise causes and consequences.

## Discussion and Conclusion

IPD-MA is a challenging process. Data collection requires considerable efforts relative to much simpler literature-based meta-analysis and the data analysis relies on multiple techniques to address the questions of heterogeneity, associations, and validation, which are all closely connected. As it is possible to correctly account for the specificities of the data, the team in charge of the analysis must have in-depth knowledge of the clinical context. In the last decade, the Cochrane collaboration has provided the community with an important set of tools and guidance that are structured and detailed step-by-step in a living e-book (35); even though the Cochrane has mainly encouraged literature-based MA, numerous tools are directly applicable to IPD-MA. In particular, the results must be carefully discussed bearing in mind their potential limitations (the risk of biases): incomplete data collection, identification of sponsors who refuse to share data, lack of standardisation, quality assessment of the various trials, etc. The field of psychiatry is particularly in need of such techniques/initiatives. For example, the question of the heterogeneity of patients with the same diagnosis and the search for subgroups and their variability in the response to treatment is central in day-to-day care and can only be explored through large heterogeneous samples with a sufficiently long follow-up. Similarly, only large samples, provided by the aggregation of data from multiple studies, allow sufficiently powerful exploration of the mediating effects of treatment efficacy, which would bring us closer to personalised medicine. Strong collaborations between all stakeholders can improve the efficiency of such a process. Thus, far, most meta-analyses have been conducted by relatively isolated teams and have often been a one-shot exercise. However, the complexity of the questions in psychiatry calls for much greater follow-up. In a recent series of position papers, Ravaud et al. (65) highlighted the need for an “ecosystem” that encourages all stakeholders (funders, trialists, journal editors, institutions, etc.) to continuously work together. Questions of interest are prioritised by the community, which also favours data sharing to considerably shorten timelines. Statisticians and physicians synthesise the data, explore the source of heterogeneity, and identify or validate the subgroups of patients that are the most likely to benefit (or who do not benefit) from an intervention. Results are discussed at regular meetings with the primary research community and reported to the various stakeholders, who in turn propose guidelines and recommendations for the treatment and care of patients. Next, and importantly, this community is in charge of keeping the synthesis up to date over time, with new evidence being added to the previous meta-analysis to refine the first results or detect improvements. Moffa et al. updated an IPD meta-analysis on the efficacy and predictors of treatment outcomes of transcranial direct current stimulation (tDGS) for major depressive disorder and how the overall results for predictors of treatment outcomes were modified by seven new trials (66). Four years separate the two publications, and an intermediate update might have brought additional and valuable information. Living meta-analyses also strengthen both collaborations and data sharing, making the process smoother and less time consuming. However, living meta-analyses require immense resources as those developed around data synthesis from trials in COVID-19 patients (67) and are almost impossible to carry out using IPD. The living meta-analysis has numerous common features with prospective meta-analyses in which the randomised clinical trials are designed with the intent to be eventually meta-analysed. A recent success in colo-rectal cancers was the IDEA project in which six trials were launched with shared structure in various countries (68). Tierney et al. have recently questioned the timing of meta-analysis update (69); in the FAME initiative, they proposed to design prospective meta-analyses, to perform systematic review of ongoing trials and and to evaluate the optimal timepoint at which new evidences may emerge and entail a modification in the standard of care. The various authors, principal investigators, sponsors are then prospectively contacted to set up the meta-analysis or its update. The field of clinical research for the evaluation of new treatments and new interventions is conducive to the development of such an ecosystem, as well-structured and stable collaborative groups often perform randomised clinical trials. International medical societies (such as the World Psychiatry or American Psychiatric Association) can play a central role in favouring the emergence of such communities. The clinical implications and unanswered questions from current meta-analyses are important for designing future trials.

All the gathered data would then be used for a living disclosure of the quality and transparency of reporting. Research transparency would likely have a positive effect and help motivate actors to develop quality improvement programs. Similarly, mapping of the stakeholders who make their data publicly available would be useful. Important efforts have been made by certain journals and health authorities, such as the National Institute of Health and the Medical Research Council, to convince, facilitate, and organise data sharing. We need a public repository to store and make fully anonymised clinical data available. The major collaboration that has been set up for genomic data is an example of how the entire community benefits from open science. An organised community would make data sharing real for the benefit of patient care and foster the spread of innovations and research findings.

## Data Availability Statement

The original contributions presented in the study are included in the article/Supplementary Material, further inquiries can be directed to the corresponding author/s.

## Ethics Statement

Ethical review and approval was not required for the study on human participants in accordance with the local legislation and institutional requirements. The patients/participants provided their written informed consent to participate in this study.

## Author Contributions

NY and XP have participated in all steps from the design of this review to the writing and approval of all materials. L-AC has participated in the data analysis, data review, writing, and approval of this manuscript. All authors contributed to the article and approved the submitted version.

## Conflict of Interest

The authors declare that the research was conducted in the absence of any commercial or financial relationships that could be construed as a potential conflict of interest.

## Publisher's Note

All claims expressed in this article are solely those of the authors and do not necessarily represent those of their affiliated organizations, or those of the publisher, the editors and the reviewers. Any product that may be evaluated in this article, or claim that may be made by its manufacturer, is not guaranteed or endorsed by the publisher.
