# Corrigendum: Reading, Conducting, and Developing Systematic Review and Individual Patient Data Meta-Analyses in Psychiatry for Treatment Issues

**DOI:** 10.3389/fpsyt.2021.789410

**Published:** 2021-11-09

**Authors:** Nadia Younes, Laurie-Anne Claude, Xavier Paoletti

**Affiliations:** ^1^Université Versailles Saint Quentin, Université Paris Saclay, CESP, Team DevPsy, Villejuif, France; ^2^Centre Hospitalier Versailles, Service Hospitalo-Universitaire de Psychiatrie de l'Adulte et d'Addictologie, Le Chesnay, France; ^3^UFR Sciences de la Santé S Veil, Université Versailles Saint Quentin, Paris Saclay, Gif-sur-Yvette, France; ^4^Institut Curie, Biostatistics, Team Statistical Methods for Precision Medicine, St Cloud, France; ^5^INSERM U900, Statistical Methods for Personalised Medicine Team (STAMPM), St Cloud, France

**Keywords:** synthesis, mediation, heterogeneity, personalised psychiatry, intervention—behavioural

In the published article, there was an error in the author list. The authors' names and surnames were exchanged. The correct author list appears below.

Nadia Younes^1,2*^, Laurie-Anne Claude ^1,2^ and Xavier Paoletti^3,4,5^

In the published article, there was an error regarding the affiliation**s** for Nadia Younes. As well as having affiliations 1 and 2, they should also have affiliation 3.

The authors apologize for these errors and state that this does not change the scientific conclusions of the article in any way. The original article has been updated.

## Publisher's Note

All claims expressed in this article are solely those of the authors and do not necessarily represent those of their affiliated organizations, or those of the publisher, the editors and the reviewers. Any product that may be evaluated in this article, or claim that may be made by its manufacturer, is not guaranteed or endorsed by the publisher.

