# Supplementary material for: Reading, Conducting, and Developing Systematic Review and Individual Patient Data Meta-Analyses in Psychiatry for Treatment Issues
Source: Front Psychiatry. 2021 Jul 29;12:644980. doi: 10.3389/fpsyt.2021.644980 (PMC8360265; doi:10.3389/fpsyt.2021.644980)
Supplement: Supplementary file 1 [file Data_Sheet_1.docx]

Supplementary material

1. Two examples of literature search
2. *Example for pharmacological treatments:*

Samara MT and al. [1] conducted an IPD-MA to examine the influence of baseline severity on the efficacy of olanzapine for the treatment of manic or mixed episodes in patients with bipolar I disorder. They described their method. Before the study, they « searched PubMed from inception to July 1, 2017, for individual patient data meta-analyses that examined the influence of initial severity of acute mania on the efficacy of any antipsychotic versus placebo. They reported their search terms: (meta-analysis OR pooled OR post-hoc) AND (bipolar OR mania) AND (drug OR antipsychotic* OR amisulpride OR aripiprazole OR asenapine (…) OR zuclopenthixol) AND placebo. No language restriction was applied. The search yielded 306 studies. On the basis of title and abstract, they closely inspected 15 studies and their references. There was no individual patient data meta-analysis investigating the effect of baseline severity on antipsychotic efficacy for bipolar mania. There were only two aggregate meta-analyses and two subgroup analyses of pooled individual patient data, but none identified a relationship between initial severity and drug–placebo difference. These studies used study-level or group-level aggregated data and had limitations such as the risk of ecological fallacy. An individual patient data meta-analysis was thus justified.

They requested IPD from Clinical Study Data Request (CSDR, <https://clinicalstudydatarequest.com>) on Feb 2, 2016: they searched for randomized controlled trials that compared the efficacy of any antipsychotic drug versus placebo for the acute treatment of manic or mixed episodes associated with bipolar I disorder. They found 33 trials; 11 trials did not include an antipsychotic treatment arm; the remaining 22 all used olanzapine, but only five of them met their prespecified eligibility criteria. All included randomized controlled trials were double- blind, sponsored by Eli Lilly, and had already been published. All data were completely anonymised by the pharmaceutical companies before they accessed them ».


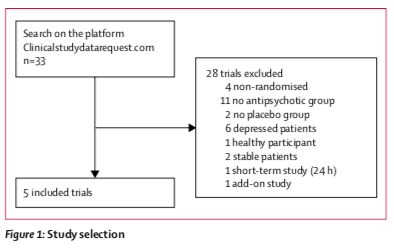


1. *Example for psychotherapies:*

Kuyken W and al [2] explored the efficacy of mindfulness-based cognitive therapy compared with usual care or active treatment, including antidepressants in the prevention of depressive relapse or recurrence in an IPD-MA.

« The study was conducted in accordance with the Preferred Reporting Items for Systematic reviews and Meta-Analyses (PRISMA) statement and the good practice guidelines of the Cochrane Collaboration IPD Methods Group. They searched for relevant publications from November 2010 (the searching end date of a previous meta-analysis to November 2014 that matched the following criteria: a. Study design: randomized trials of MBCT for the prevention of relapse in patients with recurrent major depressive disorder currently in remission and published or accepted for publication in peer-reviewed journals; b. Participants: participants aged 18 years or older, diagnosed as having recurrent major depressive disorder in full or partial remission according to a formal diagnostic classification system (major depressive disorder was defined as a diagnosis based on the DSM-III, -III-R, -IV, or -IV-TR or the International Statistical Classification of Diseases and Related Health Problems, Tenth Revision [ICD-10]); c. Intervention group: MBCT delivered according to the treatment manual; Control group: at least 1 non- MBCT treatment, including usual care; and outcome measures: number of participants meeting the diagnostic criteria for a new major depressive episode over the follow-up study period, according to accepted clinical diagnostic criteria such as the ICD-10 or the DSM-IV- TR). Studies were identified from searches of titles, abstracts, and keywords of electronic databases (EMBASE, PubMed/Medline, PsycINFO, Web of Science, Scopus, and the Cochrane Controlled Trials Register) using the following search string: (mindfulness-based cognitive therapy) OR (mindfulness based cognitive therapy) OR (MBCT) AND (depress*). No language or other limitations were imposed. They also checked reference lists of relevant studies and reviews for additional references to potentially relevant studies.

[1] M. T. Samara *et al.*, “Initial symptom severity of bipolar I disorder and the efficacy of olanzapine: a meta-analysis of individual participant data from five placebo-controlled studies,” *The Lancet Psychiatry*, vol. 4, no. 11, pp. 859–867, Nov. 2017, doi: 10.1016/S2215-0366(17)30331-0.

[2] W. Kuyken *et al.*, “Efficacy of Mindfulness-Based Cognitive Therapy in Prevention of Depressive Relapse: An Individual Patient Data Meta-analysis From Randomized Trials,” *JAMA Psychiatry*, vol. 73, no. 6, p. 565, Jun. 2016, doi: 10.1001/jamapsychiatry.2016.0076.
