# Supplementary material for: Reading, Conducting, and Developing Systematic Review and Individual Patient Data Meta-Analyses in Psychiatry for Treatment Issues
Source: Front Psychiatry. 2021 Jul 29;12:644980. doi: 10.3389/fpsyt.2021.644980 (PMC8360265; doi:10.3389/fpsyt.2021.644980)
Supplement: Supplementary file 2 [file Data_Sheet_2.DOCX]

**Reading, conducting, and developing systematic review and individual patient data meta-analyses in psychiatry for treatment issues**

Younes Nadia ^a,b^, Claude Laurie-Anne ^a,b^, and Paoletti Xavier ^c,d,e^

Supplementary material 1

To illustrate the application of the multiple guidance to conduct systematic reviews and individual patient meta-analyses, we provide several examples.

Example 1 (Evaluation of average effect):

Riper H et al. [1] conducted an IPD-MA of 19 RCTs that assessed the overall effectiveness of internet interventions for adult problem drinking in reducing alcohol consumption. The accumulated 14,198 participants, including 8,095 with post-intervention outcome data, boosted the statistical power. The primary outcome was mean weekly alcohol consumption (in standard units: SUs). Following data standardisation, the results showed an overall significant difference in mean weekly alcohol reduction in favour of the internet interventions.

Example 2 (Evaluation of average effect):

Wilkinson et al. performed an IPD-MA from ten small RCTs (resulting in N = 298 subjects) to test the efficacy of a relatively recent medication-based treatment in psychiatry on suicidal ideation: single-dose intravenous ketamine versus a control (saline placebo or midazolam). Ketamine significantly reduced suicidal ideation as measured by both clinician-administered and self-reported outcome measures within one day, with a moderate-to-large effect size at all time points post-dose over one week [2].

Example 3 (Evaluation of average effect):

Kuyken et al. conducted an IPD-MA from nine published RCTs (N = 1,258 patients) on the efficacy of Mindfulness-Based Cognitive Therapy in the prevention of depressive relapse within 60 weeks of follow-up [3]. Comparisons with active treatments indicated a reduced risk of depressive relapse over a 60-week follow-up period (hazard ratio, 0.79; 95% CI, 0.64-0.97).

Example 4 (Evaluation of average effect):

Gueorguieva et al. performed a meta-analysis of individual data from four randomised double-bind, placebo-controlled discontinuation clinical trials conducted over 26 weeks by a pharmaceutical company to understand patterns of relapse in responders to antidepressant treatment. They identified two similar relapse and two similar stable depression score trajectories for patients on active medication and for those on placebo. Active treatment significantly lowered the odds of having the “relapse” trajectory but with a limited protective effect [4].

Example 5 (Investigation of treatment effect modifiers – outcome standardization – adjusted analyses):

Furukawa et al. [5] performed an IPD-MA and an IPD-meta-regression to compare cognitive behavioural therapy, antidepressant medication, and their combination among patients with persistent depressive disorder for depression severity and dropout for any reason at 12 weeks. They were able to study several effect modifiers, such as baseline depression severity, baseline anxiety score, prior medication, age, and depression subtypes, by IPD-MA although the RCTs produced conflicting results. For example, the severity of baseline depression was identified as a predictive factor by two RCTs but not by another and the proportion of patients with prior exposure to pharmacology strongly varied between the RCTs (from 100% with prior exposure to pharmacotherapy to 24% without prior pharmacotherapy nor psychotherapy). The authors concluded that the overall average effect might be misleading, and pharmacotherapy may be a preferred option for young patients with chronic major depression because the expected dropout rate on the combination therapy was extremely high. By contrast, patients with moderate depression and mild anxiety could benefit equally well from the combination or cognitive behavioural therapy alone (which would be the preferred choice).

The authors had to convert the Montgomery – Asberg Depression Rating Scale into a 17-item HRSD using a conversion algorithm based on item response theory [5] in order to be able pool the various data.

When they studied relative treatment effects (cognitive behavioural therapy, antidepressant medication, and their combination among patients with persistent depressive disorder), they adjusted the effect for covariates assumed to be important predictive factors for the response to treatment or treatment effect modifiers [5]. Only covariates collected in all trials could be used.

The results are reported in Table S1.


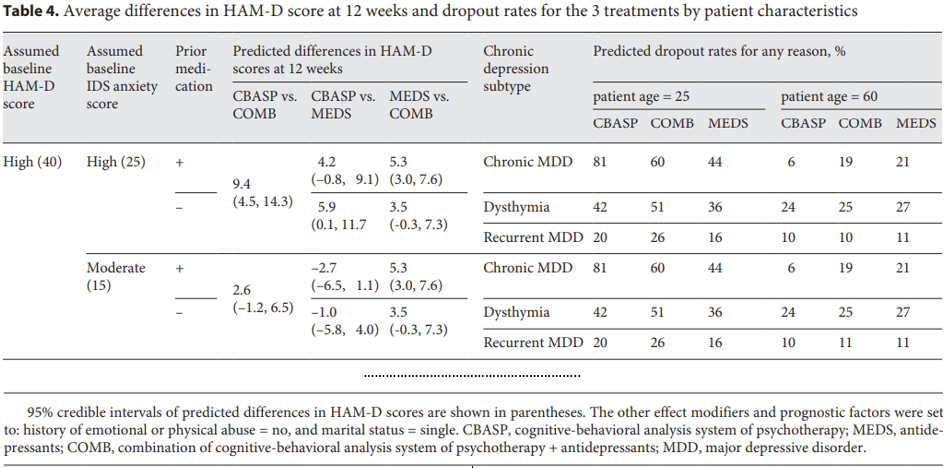


Table S1. Example of an analysis of the average differences in HAM-D score at 12 weeks and drop out rates adjusted for patient characteristics assumed to be important predictive factors for the response to treatment, reproduced from Furukawa et al. (Table 4) [5]

Example 6 (Investigation of treatment effect modifiers):

Gardner et al. aimed at assessing the difference in the effects of a parenting intervention for child conduct by IPD-MA across 13 European randomised trials (N = 1,046 families) [6]. They found no evidence for differential effects due to social disadvantage or ethnicity. This momentum is the consequence of controversial results and of the need to personalize health care.

Example 7 (Mediation Analysis):

One IPD-MA of 14 RCTs (1,070 patients) [7] explored personalised psychiatry in the field of acute phase treatments for depression by focusing on individual symptoms to identify patients who would respond to one of two effective treatments (antidepressant medication or cognitive behavioral therapy CBT) based on their pre-treatment symptomatology. Conventional MAs indicated that their efficacy (focusing on overall depression severity) is comparable and that antidepressant medication is slightly more effective. The IPD-MA showed that improvements were larger for medication than for CBT for five pre-treatment symptoms (“depressed mood”, “feelings of guilt”, “suicidal thoughts”, “psychic anxiety”, “general somatic symptoms) but not for the 12 others. Mediation analysis was performed to understand how these symptoms improved and the direct and indirect symptom-specific effects of antidepressant medication vs. CBT were estimated.

However, as shown in Figure S1, they had to assume a direct connection between an intervention and a change in a particular symptom [7]. If the treatment condition is connected to a particular symptom via one or more changes in other symptoms, it may be interpreted as an indirect symptom-specific effect. The previously identified symptom-specific effects on “feelings of guilt”, “suicidal thoughts”, “psychic anxiety”, and “general somatic symptoms” were, at least partially, direct, indicating that the larger improvements for antidepressants relative to CBT could not be fully explained by any of the other direct or indirect symptom-specific effects. The symptom-specific effect on “depressed mood” was fully indirect, suggesting that improvements in the four symptoms that were directly affected by medication relative to CBT resulted, both directly and indirectly, in a larger improvement in “depressed mood”.


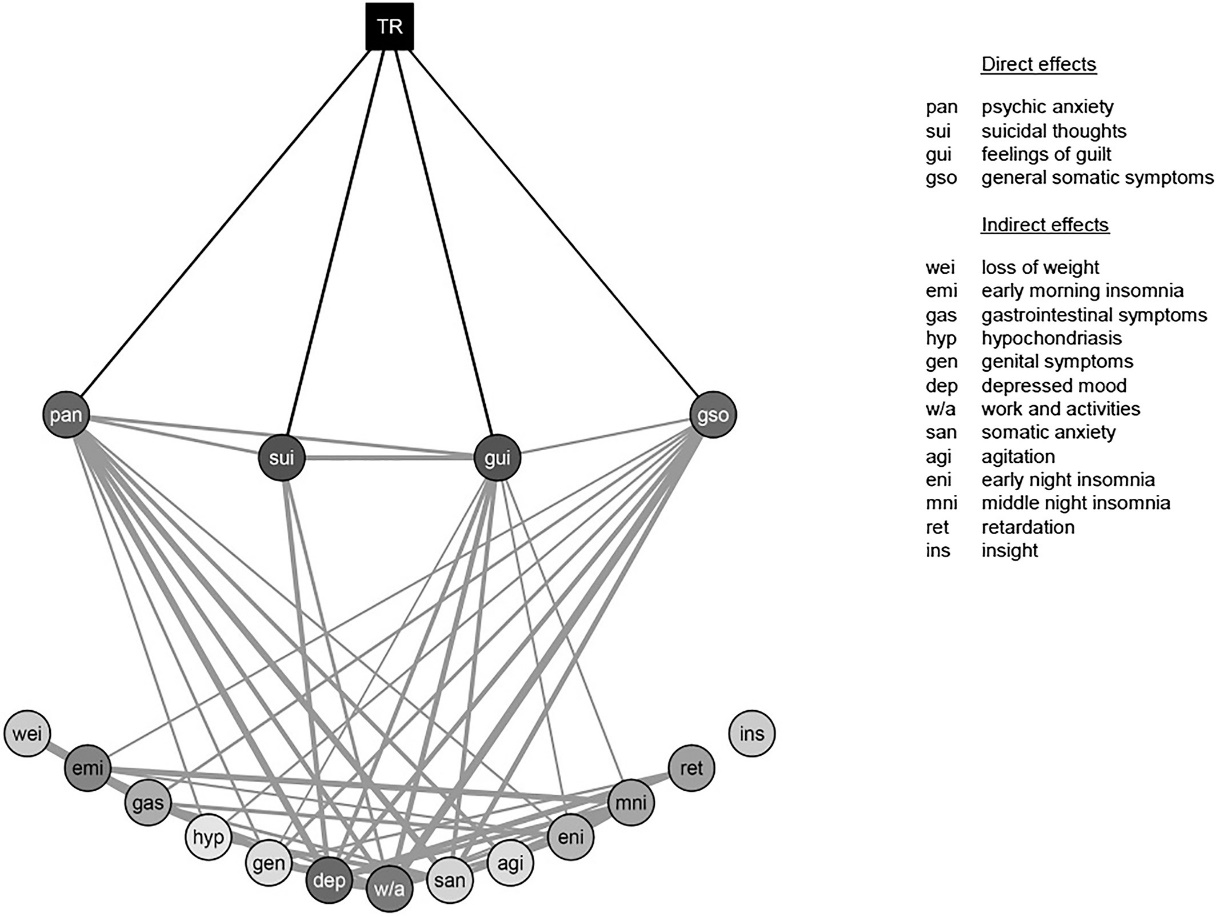


Figure S1. Example of direct acyclic graphs illustrating a direct connection between an intervention and a change in a particular symptom and the connection between various symptoms to prepare a mediation analysis of the effect of antidepressant medications. Reproduced from Boschloo et al. [7] (Figure 1).

Example 8 (Surrogacy validation):

In a statistical communication, Alonso et al. applied the IPD meta-analytical approach to validate surrogate endpoints on five randomised clinical trials (556 patients) of risperidone versus placebo for the treatment of schizophrenia at doses of 4 to 6 mg for a duration of 4 to 8 weeks [8]. They considered the positive and negative syndrome scale (PANSS) and the clinician’s global impression (CGI) as a clinical measure of change. Overall, the treatment improved both PANSS and CGI. Although there were no natural surrogates or final endpoints, the authors explored whether the treatment effect on PANSS, as assessed at the last evaluation timepoint, predicted the treatment effect on CGI and how much of the treatment effect on CGI was explained by the treatment effect on PANSS.

Example 9 (Standardization of Intervention):

An IPD-MA on the influence of baseline severity of schizophrenia on the efficacy of antipsychotics included only RCTs that used doses indicated in US Food and Drug Administration labels or the British National Formulary [5], [9].

Example 10 (Standardization of Intervention – Population – Endpoint):

The IPD-MA that assessed the symptom-specific efficacy of antidepressant medication vs CBT required manualised CBT using cognitive restructuring as the main treatment component [7]. It included “only studies including outpatients with a primary diagnosis of a DSM-II, DSM-III, or DSM-IV depressive disorder (major depressive disorder or dysthymia), as established by a standardised diagnostic interview”[7]. The primary endpoint was symptom-specific efficacy based on the most-often-used instrument for the assessment in these studies: the Hamilton Depression Rating Scale (HAM-D).

Example 11 (Standardization of the exposure):

In the IPD-MA assessing the overall effectiveness of internet interventions in reducing alcohol consumption for adults with problem drinking, alcohol consumption was standardised into standard units based on 10 grams of ethanol because the RCTs, conducted in 10 different countries (Germany, Switzerland, US, UK, Japan, Denmark, Norway, Netherlands, Canada, Sweden) differed in the quantification of alcohol in beverages based on national customs [1].

Example 12 (dealing with missing data):

In the IPD-MA investigating the efficacy of drug treatment for acute mania across geographic regions at the three-week post-baseline assessment, missing data on the outcome for week three was imputed by the measure at week four or the “last observation was carried forward” [10].

Example 13 (Pooled estimate and forest plot):

In an IPD-MA evaluating the overall efficacy of brief motivational interventions (BMIs) on reducing alcohol consumption for college students, Huh et al. estimated the size of the intervention effect and its confidence interval using a random-effect model [11]. As the distribution of alcohol consumption was strongly skewed, with many patients drinking no alcohol at all, an appropriate zero-inflated model was built, as only PDIs can allow this to be accounted for. The results, displayed in a Forest Plot (Figure S2), indicated that the intervention was not statistically significant in all but one trial (study 16). Overall, the meta-analysis demonstrated that the BMIs were associated with non-significant reductions in the probability of any drinking (OR = 0.79, 95% CI [0.61, 1.10] and the quantity drunk by participants who drank (RR = 0.96, 95% CI [0.91, 1.00]. The high statistical power obtained by pooling all the evidence showed that the efficacy of BMIs in reducing harmful drinking on college campuses was much smaller than believed.


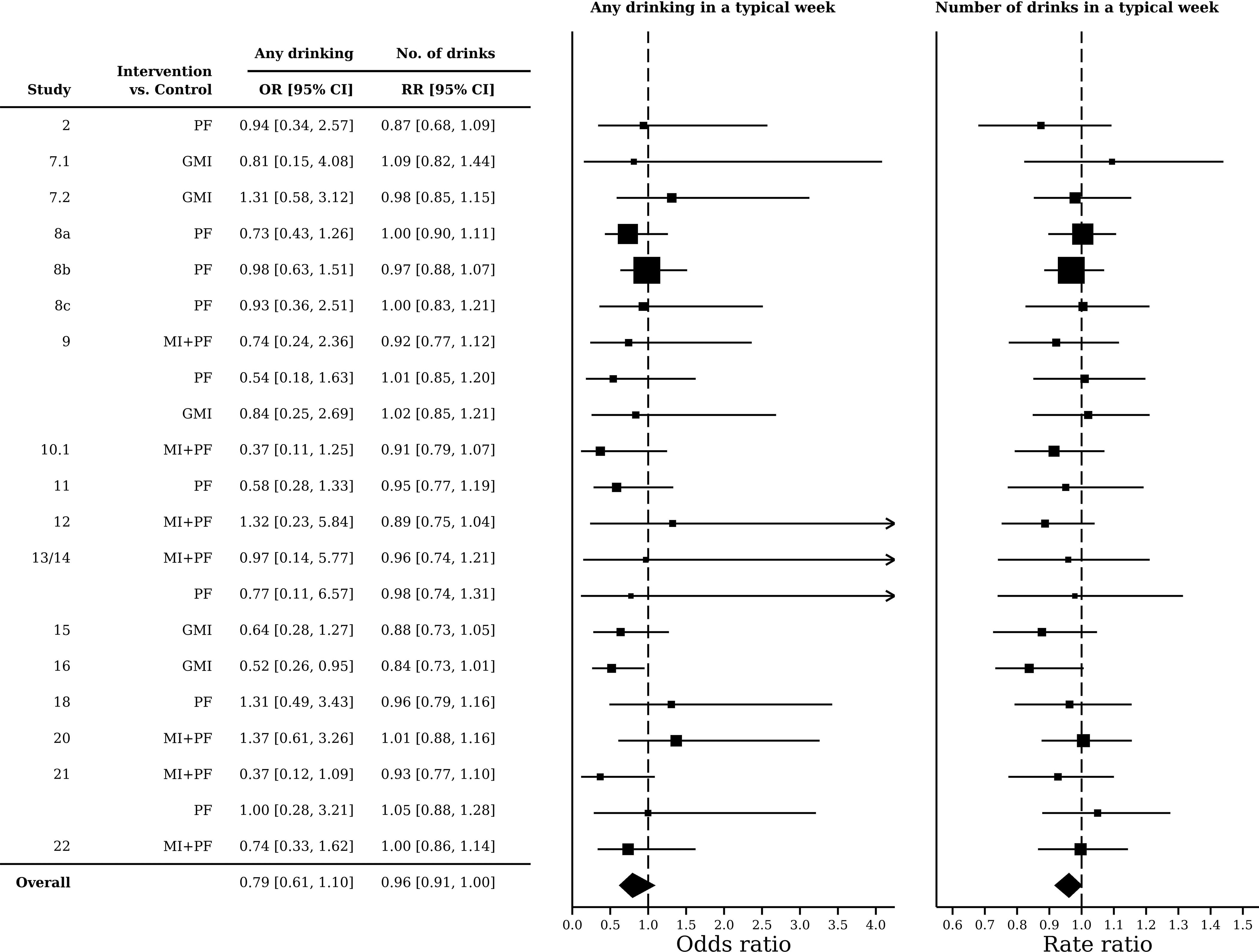


Figure S2. Example Forest Plot of the intervention effects for any drinking and the quantity of drinks when drinking, reproduced from Huh et al. (Figure 3) [11]. MI + PF: individually-delivered motivational interview with personalised feedback, PF: stand-alone personalised feedback, GMI: group motivational interview, No. of drinks: number of drinks when drinking.

Example 14 (sub-group analyses)

An IPD-MA investigated whether the baseline severity of major depression modifies the efficacy of various antidepressants [12]. They computed the Q-test, tau² and I² to explore the heterogeneity of the estimates of the interaction between treatment and baseline characteristics across the six included RCTs. There was no indication of substantial heterogeneity among the included trials (chi-squared = 7, df = 5, p = 0.017; I-squared = 35%) (cf. Figure S3).


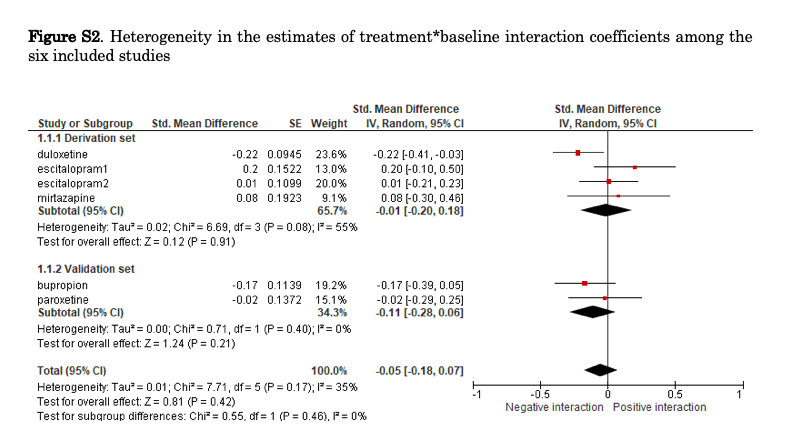


Figure S3. Example of the analysis of the efficacy of anti-depressants at the trial level in subgroups of trials defined by the baseline severity of major depression, reproduced from Furukawa et al. (Figure S2) [12]. SE = standard error. Treatment by baseline interactions were computed for each of the trials and pooled using meta-analysis technics.

References

[1] H. Riper *et al.*, “Effectiveness and treatment moderators of internet interventions for adult problem drinking: An individual patient data meta-analysis of 19 randomised controlled trials,” *PLoS Med*, vol. 15, no. 12, p. e1002714, Dec. 2018, doi: 10.1371/journal.pmed.1002714.

[2] S. T. Wilkinson *et al.*, “The Effect of a Single Dose of Intravenous Ketamine on Suicidal Ideation: A Systematic Review and Individual Participant Data Meta-Analysis,” *AJP*, vol. 175, no. 2, pp. 150–158, Feb. 2018, doi: 10.1176/appi.ajp.2017.17040472.

[3] W. Kuyken *et al.*, “Efficacy of Mindfulness-Based Cognitive Therapy in Prevention of Depressive Relapse: An Individual Patient Data Meta-analysis From Randomized Trials,” *JAMA Psychiatry*, vol. 73, no. 6, p. 565, Jun. 2016, doi: 10.1001/jamapsychiatry.2016.0076.

[4] R. Gueorguieva, A. M. Chekroud, and J. H. Krystal, “Trajectories of relapse in randomised, placebo-controlled trials of treatment discontinuation in major depressive disorder: an individual patient-level data meta-analysis,” *The Lancet Psychiatry*, vol. 4, no. 3, pp. 230–237, Mar. 2017, doi: 10.1016/S2215-0366(17)30038-X.

[5] T. A. Furukawa *et al.*, “Cognitive-Behavioral Analysis System of Psychotherapy, Drug, or Their Combination for Persistent Depressive Disorder: Personalizing the Treatment Choice Using Individual Participant Data Network Metaregression,” *Psychother Psychosom*, vol. 87, no. 3, pp. 140–153, 2018, doi: 10.1159/000489227.

[6] F. Gardner *et al.*, “Equity effects of parenting interventions for child conduct problems: a pan-European individual participant data meta-analysis,” *Lancet Psychiatry*, vol. 6, no. 6, pp. 518–527, Jun. 2019, doi: 10.1016/S2215-0366(19)30162-2.

[7] L. Boschloo *et al.*, “The symptom‐specific efficacy of antidepressant medication vs. cognitive behavioral therapy in the treatment of depression: results from an individual patient data meta‐analysis,” *World Psychiatry*, vol. 18, no. 2, pp. 183–191, Jun. 2019, doi: 10.1002/wps.20630.

[8] A. Alonso, H. Geys, G. Molenberghs, and T. Vangeneugden, “Investigating the criterion validity of psychiatric symptom scales using surrogate marker validation methodology,” *Journal of Biopharmaceutical Statistics*, vol. 12, no. 2, pp. 161–178, Jan. 2002, doi: 10.1081/BIP-120015741.

[9] S. Leucht *et al.*, “The Optimization of Treatment and Management of Schizophrenia in Europe (OPTiMiSE) Trial: Rationale for Its Methodology and a Review of the Effectiveness of Switching Antipsychotics,” *Focus (Am Psychiatr Publ)*, vol. 14, no. 3, pp. 378–386, Jul. 2016, doi: 10.1176/appi.focus.140302.

[10] C. C. Welten *et al.*, “Efficacy of drug treatment for acute mania differs across geographic regions: An individual patient data meta-analysis of placebo-controlled studies,” *J Psychopharmacol*, vol. 29, no. 8, pp. 923–932, Aug. 2015, doi: 10.1177/0269881115586938.

[11] D. Huh *et al.*, “Brief Motivational Interventions for College Student Drinking May Not Be as Powerful as We Think: An Individual Participant-Level Data Meta-Analysis,” *Alcohol Clin Exp Res*, vol. 39, no. 5, pp. 919–931, May 2015, doi: 10.1111/acer.12714.

[12] T. A. Furukawa *et al.*, “Initial severity of major depression and efficacy of new generation antidepressants: individual participant data meta-analysis,” *Acta Psychiatr Scand*, vol. 137, no. 6, pp. 450–458, 2018, doi: 10.1111/acps.12886.
